# *n*-3 Polyunsaturated Fatty Acids and Sarcopenia: Recent Advances and Mechanistic Research

**DOI:** 10.3390/nu18111660

**Published:** 2026-05-22

**Authors:** Haoran Li, Wenlong Xu, Yingjia Hu, Yi Hu, Tao Li, Rengfei Shi

**Affiliations:** School of Health and Exercise, Shanghai University of Sport, Shanghai 200438, China; 18317155933@163.com (H.L.); xwl5111@163.com (W.X.); huyingjia2929@163.com (Y.H.); huyiiii2022@163.com (Y.H.); 15735575756@163.com (T.L.)

**Keywords:** *n*-3 PUFAs, sarcopenia, muscle protein synthesis, SPMs

## Abstract

Sarcopenia is an age-related syndrome characterized by the progressive loss of skeletal muscle mass, strength, and function, significantly impairing older adults’ independence and quality of life. Given their anti-inflammatory, antioxidant, and metabolic regulatory properties, *n*-3 polyunsaturated fatty acids (*n*-3 PUFAs) have emerged as a promising nutritional strategy to mitigate this muscle degeneration. This review systematically synthesizes existing evidence regarding the association between *n*-3 PUFAs and sarcopenia. To capture the relevant literature, we searched PubMed, Web of Science, CNKI, and Wanfang Data using a combination of subject headings and free-text terms. We supplemented primary search terms—such as “*n*-3 polyunsaturated fatty acids,” “omega-3 fatty acids,” “sarcopenia,” and “muscle mass”—with mechanism-related keywords like “inflammation,” “muscle satellite cells,” and “oxidative stress.” We also manually screened the reference lists of the included literature. Our inclusion criteria encompassed interventional studies, observational studies, and high-quality reviews, while excluding conference abstracts, duplicate publications, and studies with incomplete data. This review first outlines the established biological mechanisms linking *n*-3 PUFAs to the pathological progression of sarcopenia, specifically detailing how these fatty acids improve muscle satellite cell function, suppress inflammation and oxidative stress, and ameliorate metabolic disorders. Next, we critically evaluate recent clinical studies and reviews, analyzing sources of study heterogeneity such as variations in sample size, intervention dose and duration, outcome measures, and baseline participant characteristics. We also highlight current research hotspots—including specialized pro-resolving mediators (SPMs), the gut–organ axis, combined interventions, and precision nutrition strategies—while emphasizing the functional differences between EPA and DHA to guide future intervention designs. Current evidence indicates that while *n*-3 PUFA supplementation can improve muscle strength and physical performance in older adults, its effects on muscle mass remain inconsistent. Addressing key research gaps, particularly the lack of standardized core outcome measures and unclear dose–response relationships, is critical. Ultimately, future research must prioritize developing high-bioavailability formulations, conducting personalized trials based on baseline *n*-3 PUFA status, and deepening investigations into inter-organ networks to translate these nutritional insights into effective sarcopenia prevention and management strategies.

## 1. Introduction

As the global population ages rapidly, geriatric syndromes have emerged as a critical public health issue. Sarcopenia—characterized by a progressive and widespread decline in skeletal muscle mass, strength, and physical function—severely impairs older adults’ quality of life and significantly elevates the risk of falls, fractures, disability, and mortality [1]. Recent epidemiological studies estimate its prevalence at 11% to 18% among older adults in China [2,3], compared to a global prevalence ranging from 10% to 27% [4,5]. Although the pathogenesis of sarcopenia is complex—involving impaired muscle satellite cell (MuSC) function, chronic low-grade inflammation, oxidative stress, and metabolic disorders—effective interventions remain limited, with non-pharmacological approaches relying predominantly on resistance exercise [6]. However, poor compliance and concurrent underlying diseases often prevent older adults from achieving optimal preventive and therapeutic outcomes through exercise alone [7,8]. Consequently, nutritional interventions have gained traction, offering higher compliance, broader applicability, and potential synergistic effects when combined with exercise [9,10].

Among various nutritional strategies, *n*-3 PUFAs and related supplements, such as fish oil, have garnered widespread academic attention for their unique potential in preventing and treating sarcopenia [9]. Mechanistically, *n*-3 PUFAs modulate muscle metabolism through multiple pathways. They enhance insulin-like growth factor-1 (IGF-1) sensitivity by activating the mammalian target of rapamycin (mTOR) signaling pathway, which promotes muscle protein synthesis and improves MuSC proliferation and differentiation. Furthermore, *n*-3 PUFAs and their metabolites (e.g., resolvins and protectins) effectively alleviate age-related chronic low-grade inflammation and inhibit pro-inflammatory signaling pathways, such as nuclear factor-κB (NF-κB), thereby reducing muscle protein breakdown [11,12]. However, despite the gradual elucidation of these mechanisms in basic research, clinical findings remain controversial. This controversy stems largely from substantial study heterogeneity, including variations in population characteristics (e.g., baseline nutritional status and endogenous conversion rates), intervention protocols (e.g., source, dosage, duration, and concurrent exercise), and outcome measures (e.g., muscle mass, strength, and physical function). These discrepancies reduce study comparability and hinder the establishment of a unified clinical consensus [13,14,15,16,17]. To address these gaps, this article systematically reviews the epidemiological evidence linking sarcopenia and *n*-3 PUFAs, explores the molecular mechanisms governing muscle metabolism regulation, summarizes recent clinical advances, and analyzes the sources of study heterogeneity. Ultimately, this review aims to provide robust scientific evidence and guide future research directions for nutritional strategies in sarcopenia management.

## 2. Sarcopenia

### 2.1. Overview of Sarcopenia

Rosenberg first coined the term “sarcopenia” in 1989 to describe the progressive, systemic loss of skeletal muscle mass and function. In 2010, the European Working Group on Sarcopenia in Older People (EWGSOP) defined it as an age-related syndrome characterized by a reduction in muscle mass, accompanied by decreased muscle strength or physical performance [1]. Subsequently, the 2019 EWGSOP update established low muscle strength as a primary screening indicator, requiring its concurrent presence with low muscle mass to confirm a diagnosis [18]. That same year, the Asian Working Group on Sarcopenia (AWGS) published diagnostic consensus guidelines tailored to Asian populations [19]. More recently, in 2024, the Global Sarcopenia Leadership Initiative (GLIS) integrated these concepts, defining muscle mass, strength, and power as core components, while identifying impaired physical function as the primary consequence [2]. Building on this, the 2025 AWGS consensus expanded the diagnostic scope to include individuals aged 50–64 and simplified the criteria, requiring only the concurrent presence of low muscle mass and strength, with physical performance now serving as the outcome measure [20].

### 2.2. Pathogenesis of Sarcopenia

Sarcopenia’s core clinical manifestations arise from multiple pathophysiological mechanisms, primarily involving three domains: impaired myosatellite cell function, chronic inflammation and oxidative stress, and metabolic disorders.

#### 2.2.1. Decreased MuSC Function

Muscle satellite cells (MuSCs) serve as the key cells for skeletal muscle regeneration and repair, typically residing in a specific microenvironment beneath the myofibrillar basement membrane. Following muscle injury, these cells activate, proliferate, and differentiate into myoblasts, which repair myofibrils through fusion [21,22]. However, aging drives a decline in both the number and the proliferation and differentiation capabilities of MuSCs. Within the microenvironment, abnormal activation of the Wnt signaling pathway and impaired autophagy further compromise regenerative capacity, forming a fundamental basis for sarcopenia [23,24,25,26,27]. Therefore, modulating these dynamic microenvironmental changes is crucial for maintaining muscle mass in older adults [28,29].

#### 2.2.2. Chronic Inflammation and Oxidative Stress

Patients with sarcopenia typically exhibit chronic low-grade inflammation, characterized by elevated circulating levels of pro-inflammatory factors (such as IL-6 and TNF-α). These factors promote muscle protein degradation by activating the NF-κB pathway to upregulate MuRF1 and Atrogin-1 [30,31,32,33]. Concurrently, oxidative stress directly damages muscle fibers via reactive oxygen species (ROS) and activates catabolic pathways [34]. More importantly, inflammatory factors promote ROS production, while ROS, in turn, exacerbate the inflammatory response [35,36]. This vicious cycle suppresses muscle protein synthesis and enhances degradation, ultimately driving sarcopenia progression.

#### 2.2.3. Metabolic Disorders

Metabolic dysregulation serves as another major driver of sarcopenia. Aging disrupts muscle protein metabolism, creating a dual imbalance of reduced synthesis and increased degradation. Specifically, anabolic resistance leads to decreased mTORC1 activity and impaired protein synthesis, while the abnormal activation of the ubiquitin–proteasome and autophagy–lysosomal systems accelerates protein degradation [37,38]. Furthermore, factors like mitochondrial dysfunction, insulin resistance, diminished GH/IGF-1 axis function, and declining sex hormone levels collectively form a complex network of metabolic imbalances [39,40,41].

Additionally, miRNA dysregulation contributes to these pathological processes. For example, abnormal changes in muscle-specific miRNAs (such as miR-1, miR-133, and miR-206) and the inflammation-associated miR-146a closely correlate with myosatellite cell functional decline, increased inflammation, and metabolic disorders [42]. Ultimately, these three pathophysiological domains are deeply intertwined: inflammation and oxidative stress accelerate satellite cell senescence and metabolic abnormalities, while metabolic resistance and impaired regenerative function further exacerbate inflammation and catabolism. Together, they drive the progressive loss of skeletal muscle mass and function [43,44].

### 2.3. Prevention and Treatment Strategies for Sarcopenia

Although pharmacological research is advancing to include more drugs [45], clinical management of sarcopenia primarily relies on non-pharmacological strategies, specifically exercise and nutrition [6,46]. Recently, studies increasingly focus on combining these two approaches, which demonstrates synergistic effects superior to single interventions [9,47].

#### 2.3.1. Exercise Intervention

Exercise remains the cornerstone of sarcopenia prevention and treatment. Resistance training provides the strongest evidence for improving muscle strength and function, whereas aerobic exercise yields limited effects alone; thus, multi-component interventions prove more effective [20,48,49,50]. However, underlying diseases and decreased mobility often reduce exercise adherence in older adults. Even alternative methods, such as home resistance band training or electrical muscle stimulation, show unclear long-term effects [8,50]. Consequently, researchers now emphasize more accessible nutritional interventions and combined exercise-nutrition strategies [51,52].

#### 2.3.2. Nutritional Intervention

As a crucial supplement to exercise, nutritional interventions offer particular value for elderly patients who face malnutrition risks or cannot exercise adequately.

Protein and amino acid supplementation currently represent the most well-supported nutritional strategies. Meta-analyses recommend a protein intake of 1.0–1.2 g/kg/day for older adults, significantly exceeding general adult recommendations. Specifically, leucine attracts considerable attention for its key role in stimulating muscle protein synthesis. Because whey protein contains abundant leucine and absorbs rapidly, it serves as an excellent protein source [53,54]. Notably, protein supplementation alone can effectively improve muscle mass and strength in older adults, even without concurrent exercise [55].

As a key substrate in muscle energy metabolism, creatine supports high-intensity contractions by increasing phosphocreatine stores. Supplementing creatine (typically 5 g/day) alongside resistance training effectively increases lean body mass and muscle strength in older adults, while standalone supplementation may also yield some muscle mass gains. Furthermore, creatine may protect muscles by reducing inflammation and inhibiting protein breakdown [56].

Vitamin D also plays an important role in managing sarcopenia. Because its deficiency closely correlates with decreased muscle function and increased fall risks, vitamin D supplementation actively helps improve muscle function in older adults [57,58].

Recently, *n*-3 PUFAs have attracted widespread attention for their multiple properties, including anti-inflammatory effects, mitochondrial function improvement, and enhanced protein synthesis [12,59,60]. One study suggests that supplementing over 2.5 g/day of *n*-3 PUFAs benefits sarcopenia patients by improving muscle strength and function [61]. However, clinical findings regarding their efficacy remain controversial. Significant heterogeneity across study populations, intervention protocols, and outcome indicators necessitates further systematic reviews [61,62,63].

Other nutrients, such as β-hydroxy-β-methylbutyric acid and taurine, also improve muscle function in some studies, though current evidence remains limited [64,65].

#### 2.3.3. Combined Intervention

Given the multifactorial pathogenesis of sarcopenia, single interventions often fail to achieve optimal outcomes, prompting a research shift toward combined exercise and nutrition protocols.

The 2025 AWGS consensus explicitly recommends a multimodal strategy—combining resistance training with nutritional supplementation—while placing bone health at the core of healthy longevity [20]. Furthermore, a network meta-analysis of 96 studies and 7596 participants provided high-quality evidence demonstrating that multimodal exercise (resistance plus balance training) combined with protein supplementation serves as the most effective approach for improving muscle strength, mass, and physical function in sarcopenia patients. Specifically, this combination increased grip strength by 5.45 kg, walking speed by 0.20 m/s, SPPB scores by 3.59 points, and the skeletal muscle index by 0.95 kg/m^2^ [52].

Collectively, this evidence establishes combined exercise and nutritional interventions as the preferred strategy for comprehensive sarcopenia management.

## 3. *n*-3 PUFAs

### 3.1. Differences in Chemical Structure and Function

As essential fatty acids, *n*-3 PUFAs classify into *n*-3 and *n*-6 series based on double-bond positions. They primarily comprise three types: α-linolenic acid (ALA, 18:3), eicosapentaenoic acid (EPA, 20:5), and docosahexaenoic acid (DHA, 22:6). Varying carbon chain lengths and unsaturation degrees endow each member with distinct physicochemical properties and biological functions [11,66].

Structurally and functionally, EPA and DHA differ markedly. Because DHA possesses a longer carbon chain and more double bonds, it exerts a more pronounced impact on membrane fluidity, lipid raft structure, and signal transduction [67,68]. In vitro studies using model membranes reveal opposing EPA and DHA effects on phospholipid interactions and cholesterol distribution. Specifically, EPA helps maintain intermolecular phospholipid stacking and uniform cholesterol distribution, whereas DHA induces phospholipid disorder and promotes cholesterol self-aggregation. In equimolar mixtures, these effects largely cancel each other out, underscoring their distinct and even antagonistic regulatory roles in membrane biology [68,69,70,71]. Regarding tissue distribution, DHA serves as a core phospholipid component in brain and retinal membranes, while EPA mainly regulates inflammation and acts as a precursor to specialized pro-resolving mediators (SPMs). Furthermore, these two fatty acids may compete for binding sites in membrane phospholipids. They displace *n*-6 PUFAs to varying degrees, thereby affecting the synthesis profile of inflammatory mediators [11,72,73].

### 3.2. Dietary Sources, Bioavailability, and Metabolism

Dietary sources of *n*-3 PUFAs fall into marine and plant-based categories: deep-sea fish and algae provide EPA and DHA, whereas flaxseeds and walnuts provide ALA. Notably, fish cannot synthesize EPA and DHA; instead, marine microalgae produce these fatty acids. Plankton ingest the microalgae, transferring the compounds along the food chain until they accumulate in deep-sea fish [66,74,75]. Recently, algal oil DHA supplements produced by directly fermenting microalgae have emerged as important commercial products, providing a vegetarian alternative [76]. Commercially available fish oil supplements primarily exist in two forms: triglyceride (natural) and ethyl ester (concentrated). Triglyceride-type fish oil typically exhibits higher absorption rates than the ethyl ester-type. Although krill oil offers higher bioavailability of phospholipid-type EPA and DHA, current evidence remains inconsistent and requires further verification [77,78].To utilize ALA, the body must enzymatically convert it first to EPA via Δ6-desaturase, elongase, and Δ5-desaturase, and then to DHA. However, this conversion remains extremely inefficient: humans convert approximately 5% of ALA into EPA and less than 0.5% into DHA [79,80]. Adult men convert less than 0.1% of ALA to DHA, whereas women can convert up to 9% because estrogen upregulates enzyme activity [81]. In older men, this conversion rate declines further [82]. Factors influencing conversion efficiency include sex, age, dietary *n*-6/*n*-3 ratio, disease states, and FADS/ELOVL gene polymorphisms, which encode the desaturase and elongation enzymes required for this synthesis [83,84,85]. Therefore, direct EPA and DHA supplementation proves more effective than relying on ALA conversion.

### 3.3. Basic Physiological Functions

*n*-3 PUFAs promote muscle protein synthesis and regulate mTOR and Notch signaling pathways, providing a favorable microenvironment for muscle satellite cell survival and differentiation, thereby promoting muscle growth [86]. Crucially, *n*-3 PUFAs exhibit potent anti-inflammatory and antioxidant properties. As anti-inflammatory agents, they act as competitive substrates for arachidonic acid (AA) metabolism, reducing the production of pro-inflammatory mediators such as prostaglandins and leukotrienes [87]. Furthermore, their metabolites—specialized pro-resolving mediators (SPMs)—actively promote inflammation resolution [88,89]. As antioxidants, *n*-3 PUFAs activate the NRF2/ARE signaling pathway, upregulating antioxidant enzymes including glutathione, superoxide dismutase (SOD), and catalase. By improving mitochondrial function and reducing ROS production, they alleviate oxidative stress-induced cell damage [90].

Beyond these effects, *n*-3 PUFAs regulate lipid metabolism (lowering serum triglycerides), improve insulin sensitivity, and modulate neurotransmitters [86]. These fundamental functions lay a solid foundation for their potential role in skeletal muscle health.

### 3.4. Dietary Reference Intakes and Current Population Intake

Global authoritative organizations issue varying recommendations for *n*-3 PUFA dietary reference intakes. The World Health Organization (WHO) and the Food and Agriculture Organization (FAO) recommend a daily intake of 250 mg of EPA+DHA for healthy adults [87]. The American Heart Association (AHA) recommends consuming fatty fish twice weekly (providing approximately 250–500 mg/day of EPA+DHA) [91]. The Chinese Dietary Reference Intakes (2023 edition) recommends 250–2000 mg/day of EPA+DHA and 0.6% E of ALA (approximately 1.6 g/day). Although regional differences in recommended intakes reflect varying interpretations of evidence regarding *n*-3 PUFA health benefits, all guidelines emphasize the importance of EPA and DHA intake.

However, actual global population intakes generally fall below recommended levels. Modern dietary patterns increase intake of plant oils rich in ALA but lack sufficient deep-sea fish consumption, resulting in a widespread intake gap for long-chain *n*-3 PUFAs [76,87].

**Figure 1:**
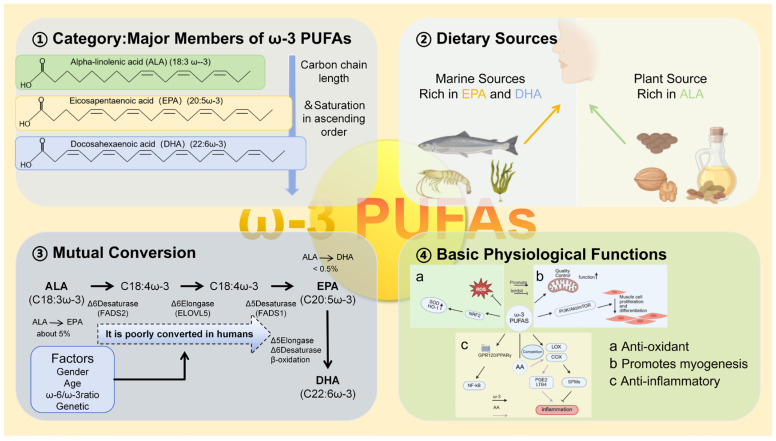
Core knowledge diagram of *n*-3 PUFAs. It presents their classification (ALA, EPA, DHA), sources (marine and plant-based), transformation (conversion of ALA to EPA/DHA is extremely inefficient), and physiological functions (anti-inflammatory, antioxidant, and metabolic regulation). Arrows and dashed lines indicate transformation pathways. The skeletal muscle background highlights their potential applications in muscle health.

Consequently, *n*-3 PUFAs increasingly attract attention from the public and academia (Figure 1 summarizes this chapter). Researchers extensively study and utilize them in sarcopenia interventions.

## 4. *n*-3 PUFAs Improve Sarcopenia

Building upon their fundamental characteristics and metabolic profiles, *n*-3 PUFAs (such as EPA and DHA) exhibit promising therapeutic effects for sarcopenia via three main mechanisms. First, by regulating cell membrane fluidity and signal transduction, they influence MuSCs resting states, activation, and differentiation. Second, their potent anti-inflammatory and antioxidant properties directly combat the destructive microenvironment arising from chronic inflammation and oxidative stress. Third, enhancing insulin sensitivity and mitochondrial function alleviates anabolic resistance and energy crises. The following sections elaborate on the specific evidence and molecular mechanisms driving these three key pathways.

### 4.1. Mechanisms of n-3 PUFAs in Improving Sarcopenia

#### 4.1.1. *n*-3 PUFAs Improve Sarcopenia by Improving MuSC Activity

*n*-3 PUFAs directly and indirectly regulate MuSC functions.

##### Direct Regulatory Effects of *n*-3 PUFAs on MuSC Activity

Extensive evidence highlights the crucial role of *n*-3 PUFAs in regulating MuSC activity and promoting muscle regeneration. In in vitro C2C12 cell experiments, Russ DW et al. demonstrated that *n*-3 PUFAs reduced dye leakage areas following muscle membrane injury across all tested concentrations, suggesting accelerated healing via enhanced membrane resealing and repair [92]. Under combined exposure to palmitic acid (lipotoxicity) and TNF-α (inflammation), EPA partially rescued myotube formation capacity by upregulating MyoD, myogenin, IGF-II, and IGFBP-5—key factors for MuSC function [93]. Similarly, another study revealed that DHA counteracts lipotoxicity: co-incubating mouse myoblasts with palmitate and DHA induced myotube hypertrophy, preventing the significant cell atrophy caused by palmitate alone [94]. S. C. de Carvalho et al. observed that *n*-3 PUFAs reduce MMP-9 gene expression and improve MuSC transplantation, activation, and muscle regeneration, partially via macrophage-dependent mechanisms [95]. Furthermore, EPA enhances skeletal muscle satellite cell proliferation and differentiation by activating phosphoglycerate mutase 2 (PGAM2), an enzyme catalyzing the conversion of 2-phosphoglycerate to 3-phosphoglycerate in glycerol metabolism. This activation triggers the PI3K/AKT signaling pathway, thereby promoting skeletal muscle growth and regulating glucose metabolism [96].

##### Indirect Regulation of MuSC Activity by *n*-3 PUFAs

Because myosatellite cell differentiation relies heavily on energy from mitochondrial oxidative phosphorylation, oxidative stress and inflammation significantly influence satellite cell fate. *n*-3 PUFAs indirectly support myosatellite cell activity and differentiation by improving mitochondrial function and alleviating both oxidative stress and inflammation. Crucially, the mTOR signaling pathway serves as a key hub for *n*-3 PUFAs to regulate muscle protein metabolism. Specifically, *n*-3 PUFAs stimulate anabolism by activating the mTORC1 complex, phosphorylating downstream kinases, and promoting protein synthesis [12]. Additionally, *n*-3 PUFAs finely regulate this pathway at the membrane level; for instance, DHA alters the structure of phosphatidylinositol 4,5-bisphosphate (PIP2), reducing its affinity for PI3K and inhibiting Akt hyperphosphorylation [97]. Furthermore, reviews of in vitro studies highlight the myoblast-level protective mechanisms of EPA and DHA. In lipopolysaccharide (LPS)-induced inflammatory environments, EPA protects mTOR phosphorylation by inhibiting NF-κB and AP-1 transcription factors, thereby maintaining muscle protein synthesis. Similarly, in adipose tissue, the mTORC2 complex mediates fish oil-induced improvements in systemic insulin sensitivity [98]. Moreover, the flavonoid-DHA complex FLAVOmega β comprehensively enhances mitochondrial function, stimulates angiogenesis, and improves muscle fatigue resistance by inhibiting inflammation and fibrosis, reducing reactive oxygen species (ROS), and promoting muscle fiber transformation into an oxidative metabolic phenotype [99].

Resolvin D2, an *n*-3 PUFA-derived lipid mediator, promotes muscle regeneration and functional recovery in injury or muscular dystrophy models. In vitro, it enhances slow-twitch myosin heavy chain myotube formation. In vivo, it increases muscle strength and fiber size, while regulating fiber type distribution in damaged muscles (e.g., transforming slow-twitch gastrocnemius to type I and tibialis anterior to type IIB). Notably, it lacks these effects in undamaged tissues, implying synergistic interactions with other factors to determine muscle fiber types [100]. Finally, the Notch signaling pathway critically regulates muscle satellite cell resting maintenance, post-injury expansion, fusion, and self-renewal, although its activity declines with age. Previous reviews suggest that *n*-3 PUFAs may influence satellite cell fate determination by regulating Notch and other signaling pathways; however, lacking direct experimental evidence necessitates further investigation [94].

In summary, *n*-3 PUFAs foster a favorable microenvironment for MuSC survival and differentiation through multiple indirect pathways. By enhancing mitochondrial function, mitigating oxidative stress, producing pro-repair lipid mediators, and modulating anti-inflammatory, anti-lipotoxic, mTOR, and Notch signaling, these fatty acids robustly support muscle regeneration.

#### 4.1.2. *n*-3 PUFAs Improve Sarcopenia by Inhibiting Chronic Inflammation and Oxidative Stress

By deploying dual anti-inflammatory and antioxidant mechanisms, *n*-3 PUFAs target the two core contributors to sarcopenia—chronic low-grade inflammation and oxidative stress. They inhibit myoprotein degradation, alleviate mitochondrial damage, and effectively mitigate the pathological progression of sarcopenia.

##### *n*-3 PUFAs Directly Inhibit Inflammation and Oxidative Stress

Regarding anti-inflammatory effects, *n*-3 PUFAs directly suppress inflammatory responses through multiple pathways. One well-understood mechanism involves inhibiting the NF-κB signaling pathway, which reduces pro-inflammatory cyclooxygenase production and downregulates atrophy-related genes like muscle ring finger protein 1 (MuRF-1), ultimately inhibiting the ubiquitin-proteasome system [12]. Furthermore, *n*-3 PUFAs bind to G protein-coupled receptors (GPCRs) and activate peroxisome proliferator-activated receptors (PPARs), thereby inhibiting NF-κB activation and reducing the expression of inflammatory factors such as IL-6 and TNF-α [66]. Specifically, EPA and DHA supplementation enhances free fatty acid receptor 4 (FFAR4/GPR120) activation, reduces serum levels of TNF-α, IL-6, and IL-18, and increases levels of the anti-inflammatory cytokine IL-10 [101]. As competitive substrates for AA metabolism, EPA and DHA also reduce the production of pro-inflammatory mediators, including prostaglandins and leukotrienes [11]. Additionally, ALA reduces inflammatory cell infiltration and tissue damage by targeting the JAK1-NOTCH1/2 signaling pathway and downregulating the MCP-1/CCR2 axis [102]. Finally, *n*-3 PUFA metabolites—SPMs (including resolvins, protectins, and maresins)—actively promote inflammation resolution by inhibiting IκB phosphorylation and degradation. This blockade prevents NF-κB nuclear translocation and downregulates pro-inflammatory cytokines such as TNF-α, IL-6, IL-8, and IL-12 [88,89].

Regarding antioxidant activity, *n*-3 PUFAs directly activate antioxidant defense mechanisms. Recent reviews highlight that *n*-3 PUFAs activate NRF2-mediated antioxidant responses. Because NRF2 regulates antioxidant enzymes like glutathione, superoxide dismutase (SOD), and catalase, its activation enhances cellular reactive oxygen species (ROS) scavenging and mitigates oxidative stress-induced muscle damage [103]. Animal studies further confirm that *n*-3 PUFAs enhance antioxidant enzyme activity [104]. Furthermore, *n*-3 PUFA-rich walnut oil alleviates rotundone-induced ROS increases. Although it may not lower baseline ROS levels, this finding confirms *n*-3 PUFAs’ specific protective role under oxidative stress [105]. Similarly, in mice with chronic heart failure, *n*-3 PUFA supplementation reduced the oxidized-to-total glutathione ratio, decreased mitochondrial ROS production, restored ATP synthesis, and prevented muscle mass loss [106].

##### Indirect Regulation of Inflammation and Oxidative Stress by *n*-3 PUFAs

Beyond direct effects, *n*-3 PUFAs indirectly alleviate inflammation and oxidative stress by correcting metabolic dysregulation. Because mitochondrial dysfunction drives excessive ROS production in sarcopenia and underpins energy metabolism disorders, improving mitochondrial function and reducing oxidative stress constitute key mechanisms for *n*-3 PUFAs to promote muscle health [107]. Studies indicate that *n*-3 PUFAs exert both anti-inflammatory and metabolic regulatory effects at the mitochondrial level by downregulating oxidative phosphorylation activity, thereby reducing IL-1β production triggered by polysaccharide metabolism [108].

The excessively high *n*-6/*n*-3 PUFA ratio in modern diets (up to 10:1 in Western diets) promotes inflammation and excessive ROS production. Correcting this ratio via *n*-3 PUFA supplementation helps reduce ROS production and improve the overall metabolic environment. Corroborating this, a lower *n*-6 (linoleic acid)/*n*-3 (EPA) ratio promotes apoptosis and lowers ROS levels [109]. Furthermore, *n*-3 PUFA supplementation promotes the proliferation of beneficial bacteria (e.g., *Lactobacillus*, *Bifidobacterium*, and butyrate-producing bacteria), improves dysbiosis, alleviates systemic inflammation, and enhances its own absorption rate [110,111], reflecting a synergistic relationship between metabolic improvement and inflammation regulation. Concurrently, *n*-3 PUFAs modulate apoptotic pathways. For example, under iron overload, they dynamically regulate ROS by upregulating pro-apoptotic genes (e.g., Caspase-8 and p53) and downregulating the anti-apoptotic gene BCL2, thereby promoting overall cellular adaptation to metabolic stress [112].

Chronic inflammation and oxidative stress form a vicious positive feedback loop in sarcopenia: inflammatory factors stimulate excessive ROS production, while ROS further exacerbate inflammatory factor expression, accelerating muscle protein degradation and functional decline. *n*-3 PUFAs act simultaneously on two key nodes in this loop: they inhibit NF-κB activation to reduce inflammatory mediator production while lowering ROS levels via NRF2-mediated antioxidant responses and improved mitochondrial function. This dual-target action enables *n*-3 PUFAs to break this vicious cycle at its source, restoring skeletal muscle redox balance and inflammatory homeostasis. Consequently, this mechanism inhibits myoprotein degradation, protects mitochondrial function, and provides a robust molecular basis for sarcopenia prevention and treatment.

#### 4.1.3. *n*-3 PUFAs Improve Sarcopenia by Regulating Metabolic Disorders

*n*-3 PUFAs effectively target the metabolic disorders central to sarcopenia’s pathology by mitigating mitochondrial dysfunction, correcting protein metabolism imbalances, and alleviating insulin resistance.

##### Direct Regulation of Metabolic Dysfunction by *n*-3 PUFAs

Clinically, a 6-month course of *n*-3 PUFA treatment in patients with non-alcoholic steatohepatitis (NASH) significantly improves mitochondrial function markers [113].

Regarding protein metabolism, *n*-3 PUFAs bidirectionally “promote synthesis and inhibit degradation.” Specifically, they promote muscle protein synthesis by modulating the PI3K/Akt/mTOR pathway while simultaneously inhibiting FoxO transcription factors, thereby blocking the autophagy-lysosomal and ubiquitin-proteasomal degradation systems. Furthermore, *n*-3 PUFAs enhance amino acid transport, ensuring adequate substrate availability for synthesis. For instance, in heat-stressed mice, *n*-3 PUFA supplementation alleviates structural and oxidative damage by inhibiting specific phosphorylation events in the PI3K/Akt/mTOR pathway [114]. As membrane components, *n*-3 PUFAs additionally modulate membrane-associated signaling proteins; for example, DHA alters PIP2 structure to inhibit its binding to PI3K, thereby reducing PIP3 accumulation and preventing abnormal Akt phosphorylation [97].

Concerning insulin sensitivity, *n*-3 PUFAs directly enhance insulin signaling. Notably, fish oil supplements improve insulin sensitivity via mTORC2-dependent pathways [115,116,117,118]. Moreover, *n*-3 PUFAs facilitate cellular glucose uptake by upregulating glucose transporter 1 (GLUT1) activity—an effect driven by increased membrane fluidity and PPARγ-dependent signaling pathway activation [119].

##### *n*-3 PUFAs Indirectly Protect Muscle by Improving the Metabolic Environment

Beyond direct effects, *n*-3 PUFAs indirectly protect skeletal muscle by optimizing the overall metabolic environment. Under insulin resistance, skeletal muscle glucose uptake and utilization decline. Because skeletal muscle accounts for 80% of insulin-mediated glucose disposal, sarcopenic muscle loss directly impairs systemic glucose metabolism, thereby exacerbating insulin resistance and fueling a vicious cycle [120,121]. By restoring insulin sensitivity, *n*-3 PUFAs effectively break this cycle.

Furthermore, metabolic dysfunction often involves visceral fat accumulation and pathological lipid infiltration into muscle tissue. Intramuscular adipose tissue (IMAT) deposition drives M2 macrophage polarization, enhances fibro-adipogenic precursor (FAP) signaling, and exacerbates muscle fiber damage [122]. Simultaneously, adipose-derived lipotoxic substances and leptin resistance blunt muscle insulin signaling, thereby accelerating atrophy. Sarcopenia also disrupts the crosstalk between myokines and adipokines, typically elevating myostatin and reducing adiponectin levels [123,124,125,126]. By leveraging their anti-inflammatory and antioxidant properties, *n*-3 PUFAs promote lipid metabolism and indirectly correct this imbalance.

Indirectly regulating protein metabolism, insulin resistance blunts the Akt pathway, suppressing protein synthesis and releasing FoxO transcription factors from inhibition. This release abnormally activates the autophagy and ubiquitin-proteasome systems, accelerating muscle protein degradation. Moreover, myostatin—a TGF-β superfamily member—inhibits muscle growth by suppressing myosatellite cell activity and the Akt/mTOR pathway [127]. By enhancing insulin sensitivity, *n*-3 PUFAs indirectly restore Akt/mTOR signaling and suppress FoxO activity, thus preserving protein metabolic homeostasis.

Additionally, optimizing the *n*-3/*n*-6 PUFA ratio improves gut microbiota composition and alleviates insulin resistance [115], reflecting the multidimensional link between metabolic improvement and muscle protection.

Ultimately, *n*-3 PUFAs exert dual mechanisms of “direct correction” and “indirect protection” against metabolic disorders. Directly, they improve mitochondrial respiratory function, bidirectionally regulate protein metabolism, and enhance insulin signaling. Indirectly, they maintain skeletal muscle metabolic homeostasis by correcting the *n*-6/*n*-3 ratio, reducing lipotoxic steatosis, and breaking the vicious cycle of insulin resistance and muscle loss, comprehensively delaying sarcopenia progression.

**Figure 2:**
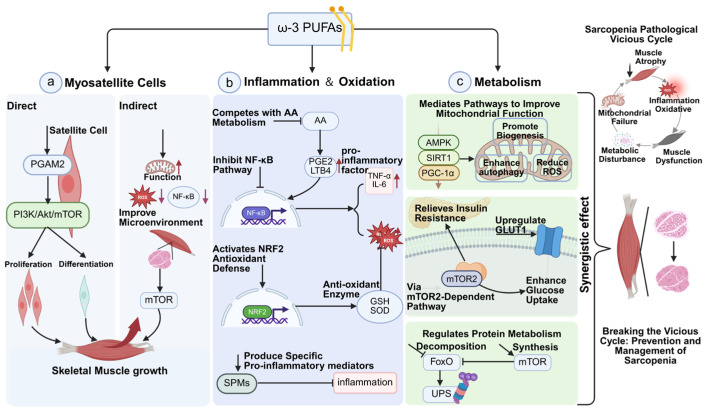
*n*-3 PUFAs improve sarcopenia through a three-level mechanism: a. At the level of muscle satellite cells, they directly or indirectly promote proliferation, differentiation, regeneration, and repair; b. At the level of inflammation and oxidative stress, they inhibit NF-κB, produce SPMs for anti-inflammation, activate NRF2 for antioxidation, and break the vicious cycle; c. At the level of metabolism, they improve mitochondrial function, alleviate insulin resistance, and regulate protein metabolism. These three levels work together to form a complex regulatory network.

In conclusion, *n*-3 PUFAs protect against sarcopenia across multiple dimensions. First, they support muscle regeneration by promoting MuSC differentiation and regulating key pathways like Notch. Second, they dismantle the chronic inflammation-oxidative stress loop by suppressing NF-κB and activating NRF2-mediated defenses. Third, they rescue muscle energy metabolism and protein homeostasis by enhancing mitochondrial quality control, regulating protein turnover, and increasing insulin sensitivity. Far from operating independently, these three intertwined and synergistic mechanisms form a robust regulatory network through which *n*-3 PUFAs ameliorate the pathological progression of sarcopenia (Figure 2).

Notably, although mechanisms have been studied in depth, existing clinical evidence remains heterogeneous. Consequently, establishing conclusive evidence regarding *n*-3 PUFA efficacy in sarcopenia prevention and treatment requires further validation through additional high-quality randomized controlled trials (RCTs).

### 4.2. Clinical Evidence for n-3 PUFAs in Sarcopenia Prevention and Treatment

The preceding sections reviewed cellular and molecular mechanisms by which *n*-3 PUFAs promote muscle satellite cell activity, improve mitochondrial function, and regulate the Notch signaling pathway. Although these mechanisms provide a theoretical basis for *n*-3 PUFA interventions in sarcopenia, translating this theory into clinical application requires answering two key questions: what actual effects do these interventions produce in human populations, and why do study results differ? This section systematically reviews the clinical evidence regarding *n*-3 PUFA effects on muscle mass and function in older adults, focusing on potential sources of research heterogeneity.

#### 4.2.1. The Interventional Effects of *n*-3 PUFAs Supplementation on Muscle Mass and Function in Older Individuals

Clinical trial results regarding *n*-3 polyunsaturated fatty acid (PUFA) supplementation in older adults with or at risk of sarcopenia remain mixed but generally positive. A 2023 systematic review analyzing 14 clinical and 11 preclinical studies found that among the 12 clinical trials using *n*-3 PUFA interventions, 8 reported positive effects on muscle, whereas 6 observed no significant effects. Notably, supplementation combined with resistance training in 7 studies demonstrated superior intervention outcomes [128].

Regarding standalone *n*-3 PUFA supplementation, some trials yield positive results. A 2026 randomized controlled trial (n = 200, elderly Chinese population, 6-month intervention, 4 g/day fish oil containing 1.34 g EPA and 1.07 g DHA) demonstrated that fish oil-derived *n*-3 PUFAs significantly increased thigh circumference, total and appendicular skeletal muscle mass, grip strength, and the time-to-stand-and-walk test [14]. Other RCTs report that standalone *n*-3 PUFA supplementation improves knee extension strength and walking speed in older adults with good mobility [129]. Similarly, Alkhedhairi SA et al. found that six months of krill oil supplementation (4 g/day) in 102 older participants increased grip strength and lateral quadriceps thickness [130]. Conversely, several trials report negative results. The large-scale, 3-year DO-HEALTH trial (n = 2157, community-dwelling healthy older adults, 1 g/day marine-derived *n*-3 PUFAs) revealed no significant effects on appendicular lean mass index (ALMI) changes or sarcopenia risk [62]. Furthermore, a 24-week RCT using standalone nutritional intervention showed no improvement in muscle strength or physical function [15]. while another 6-month study observed increased muscle mass without significant improvements in muscle strength or mitochondrial function [131].

Combining *n*-3 PUFAs with exercise or other nutritional components often yields greater benefits. A systematic review and meta-analysis by Dam et al. (9 RCTs, n = 286, ≥65 years) found that combining *n*-3 PUFAs with resistance training significantly improved sit-to-stand test performance, despite not enhancing muscle mass or neuromuscular function; however, high bias risk limited evidence quality [63]. A 2-month RCT involving 60 elderly patients with sarcopenia showed that a formula containing *n*-3 PUFAs, leucine, and specific probiotics significantly increased appendicular lean body mass and grip strength while reducing visceral fat [132]. Another 12-week intervention combining resistance training with a multi-nutrient supplement (whey protein, creatine, vitamin D, and *n*-3 PUFAs) significantly improved lean body mass, maximum strength, and fast-twitch muscle fiber cross-sectional area in community-dwelling men with sarcopenia—a synergistic effect confirmed by other studies [133]. Furthermore, intervention effects may vary by gender: one study showed that older women supplementing *n*-3 PUFAs alongside resistance training experienced significant improvements in muscle function and mass, an effect absent in men [134].

In summary, current clinical evidence does not yet support *n*-3 PUFAs supplementation as a universal sarcopenia intervention, although studies suggest its potential value for specific populations or within combined interventions. Overall, *n*-3 PUFAs effects appear context-dependent. Key outcome-influencing variables include intervention duration, baseline participant status, and supplement composition (e.g., dosage, EPA/DHA ratio, and nutrient combinations). This heterogeneity underscores the need for targeted studies to identify optimal intervention strategies and the specific populations most likely to benefit.

#### 4.2.2. Potential Sources of Heterogeneity in Research

The inconsistencies in the aforementioned findings reflect the complex, multifactorial nature of *n*-3 PUFAs effects on skeletal muscle. Systematically identifying these heterogeneity sources clarifies existing study differences and guides the optimization of future research designs.

##### Differences in Intervention Protocols and Endpoint Definitions

Determining the effective dose remains a major point of contention in clinical studies of *n*-3 PUFA interventions for sarcopenia. The commonly used dose range (typically 1–4 g/day of EPA + DHA) has yielded inconsistent conclusions, highlighting a lack of consensus. Heterogeneity in dose–response primarily stems from differences in intervention design. First, the daily supplementation dose, intervention duration (12 to 24 weeks or longer), and supplement EPA-to-DHA ratio vary widely across studies, directly impacting biological effects [14,62,130,131,134,135]. As discussed previously, EPA and DHA exhibit distinct functions in combating muscle atrophy: although both alleviate age-related declines in muscle mass and function, EPA outperforms DHA in enhancing muscle strength, providing antioxidant effects, improving mitochondrial function, and promoting protein synthesis; conversely, DHA more effectively inhibits protein degradation and increases IκBα levels [86,136]. Animal studies indicate that EPA more potently activates anabolic pathways [137], while human studies suggest gender-specific effects and potential synergistic interactions [138,139]. Consequently, optimizing the EPA/DHA ratio proves more critical than simply increasing the total dose, providing a basis for precision supplementation strategies.

Furthermore, co-supplementation with other nutrients (e.g., protein or vitamin D) may produce synergistic or antagonistic effects, complicating the interpretation of single-dose responses [13,15,129,132,133]. Additionally, diverse outcome measures across studies reduce direct comparability. Researchers varyingly assess muscle mass (e.g., appendicular lean body mass, skeletal muscle index), muscle function (e.g., grip strength, chair stand test, gait speed), molecular indicators (e.g., protein synthesis rate), or clinical endpoints (e.g., sarcopenia incidence) [140,141]. Table 1 summarizes recent RCT studies, illustrating these diverse study designs.

##### Differences in Baseline Characteristics of Subjects

Subjects’ baseline nutritional status, physical activity levels, and sex act as crucial confounding factors influencing *n*-3 PUFA intervention efficacy. Because baseline levels define the “starting point” and “potential for improvement,” individuals already maintaining high *n*-3 PUFA diets prior to intervention derive minimal marginal benefit from supplementation, often leading to “ineffective” clinical conclusions (Notably, the erythrocyte membrane *n*-3 index—the percentage of EPA and DHA in total red blood cell phospholipids—accurately reflects this tissue status over the past 2–4 months.) [16,143,144].

Conversely, *n*-3 PUFAs exhibit more pronounced efficacy in individuals with underlying health conditions or severe baseline deficiencies [17]. Interventions targeting populations with confirmed chronic inflammation, diagnosed sarcopenia, or those evaluated through challenging preclinical models typically demonstrate stronger protective outcomes. For example, while *n*-3 PUFAs do not affect baseline reactive oxygen species (ROS) levels under normal conditions, they effectively alleviate significant ROS increases induced by rotenone exposure [105]. Similarly, during heat stress, *n*-3 PUFA supplementation enhances protective autophagy by inhibiting the PI3K-Akt-mTOR pathway [114], while animal studies confirm that ALA reduces toxin-induced cell death and neuroinflammation [145]. Consequently, clinical interventions in specific comorbidities often report positive results [146,147]. Similar patterns emerge in skeletal muscle research: older adults with low baseline dietary intake or plasma levels prove more sensitive to supplementation than those with adequate baseline levels [62].

Furthermore, gender differences warrant attention. One study showed that older women supplementing *n*-3 PUFAs combined with resistance training experienced significant improvements in muscle function and quality, whereas men under identical conditions did not [134]. Similarly, Alsoowail AT et al. reported a positive correlation between *n*-3 PUFA intake and grip strength indices exclusively in older women, a discrepancy likely related to estrogen’s regulatory effects on muscle metabolism [148].

Ultimately, high results heterogeneity remains a core challenge in clinical *n*-3 PUFA research. This variance primarily stems from differences in subjects’ pre-intervention nutritional status, underlying disease severity, and sex. Therefore, future research designs must prioritize systematically assessing and reporting baseline levels. Standardizing biomarker measurements (e.g., the erythrocyte *n*-3 index) and stratifying subjects by disease characteristics constitute the cornerstone for accurately identifying target populations, elucidating dose–response relationships, and developing personalized nutritional strategies.

##### Individual Differences in *n*-3 PUFAs Metabolism

*n*-3 PUFAs possess two distinct but interrelated efficacy determinants: bioavailability and endogenous conversion rates. Bioavailability encompasses the overall efficiency of dietary digestion, absorption, lymphatic transport, hepatic metabolism, and tissue distribution. Supplement source and formulation (e.g., ethyl esters, triglycerides, phospholipids) significantly influence this metric [142,149,150]. For instance, phospholipid-rich krill oil absorbs more readily than standard fish oil [77]. Furthermore, varying manufacturing processes (such as molecular distillation and urea complexation) introduce variations in final product purity, oxidative stability, and residual contaminants, indirectly affecting intervention efficacy even within the same formulation type [151,152]. Because direct dietary sources typically provide less than 30% of the recommended 250 mg to 2 g daily EPA+DHA intake, exploring optimal supplement forms is crucial for maximizing biological functions [153].

Regarding ALA, endogenous conversion to active forms (EPA and DHA) serves as a critical rate-limiting step. This conversion depends on a Δ6-desaturase encoded by the FADS2 gene [154]. and remains highly inefficient, with overall rates typically below 10% [155,156]. Various factors tightly regulate this process, including dietary *n*-6/*n*-3 PUFA ratios (which compete for the same enzymes), sex, and age [82,157,158,159,160]. Therefore, achieving adequate LC-PUFA levels relies heavily on directly consuming pre-formed marine-derived *n*-3 PUFAs, primarily from fish and algae [161,162]. However, both sources present drawbacks: fish accumulate environmental pollutants like heavy metals [163], while algal sources typically feature low concentrations [157].

Furthermore, metabolic kinetic studies in older adults indicate that while DHA metabolism remains relatively stable post-supplementation, EPA metabolism slows. This deceleration likely results from DHA inhibiting EPA elongation or decreasing plasma clearance, suggesting compensatory metabolic regulation in the elderly [164].

In summary, current clinical evidence lacks consistent conclusions regarding *n*-3 PUFA efficacy in sarcopenia. Large-scale, high-quality trials (like DO-HEALTH) observed no significant benefits, while smaller studies reported positive effects. This heterogeneity stems from complex interactions among intervention protocols, baseline characteristics, endpoint definitions, and individual metabolic differences. Future randomized controlled trials must strictly account for these variables in subgroup analyses to clarify optimal dosages, methods, and applicable populations.

### 4.3. Research Hotspots and Future Directions of n-3 PUFAs in the Prevention and Treatment of Sarcopenia

Based on the aforementioned heterogeneity, future studies urgently need to establish standardized core outcome measures and thoroughly examine dose–response relationships (including total doses and specific component ratios). Beyond standardization, several emerging frontiers warrant focused investigation.

#### 4.3.1. SPMs

Specialized pro-resolving mediators (SPMs)—including resolvins, protectins, and maresins—represent a highly promising therapeutic frontier. As potent bioactive derivatives of *n*-3 PUFAs, these metabolites mediate their parent fatty acids’ anti-inflammatory and tissue-repairing functions; their systemic levels rise following omega-3 supplementation [88,89,157]. Recent reviews systematically map the metabolic origins, structural relationships, and target sites of various SPMs, providing an intuitive navigational framework for this complex field [165]. Mechanistically, in exercise-induced inflammation models, resolvin D1 (RvD1) upregulates anti-inflammatory markers and inhibits pro-inflammatory cytokines, likely via the BDNF/TrkB/PI3K/AKT pathway [166]. Furthermore, RvD1 directly protects muscle cells under chronic inflammatory stress [167]. Similarly, Maresin 1 (MaR1) regulates inflammatory responses via PI3K/Akt/mTOR and ERK pathways [168,169], while exhibiting antioxidant activity by scavenging ROS and inhibiting the p38/ERK/NF-κB cascade [170]. Consequently, supplementing SPMs, their stable analogs, or receptor agonists demonstrates beneficial effects in animal models of musculoskeletal diseases (MSDs), highlighting high translational potential [171,172]. Alongside natural SPMs, researchers are advancing synthetic analogs like OMT-28, an *n*-3 PUFA-derived epoxide that effectively prevents mitochondrial dysfunction while boasting high oral bioavailability [173].

#### 4.3.2. Organ Axis

*n*-3 PUFAs interact with the gut microbiota and the musculoskeletal axis through multiple pathways. Abundant evidence confirms that *n*-3 PUFAs optimize the gut microbiome [157,174], which in turn enhances the absorption of nutrients, including the PUFAs themselves. They resolve dysbiosis by promoting beneficial microbes (e.g., *Lactobacillus*, *Bifidobacterium*) and increasing short-chain fatty acids (SCFAs) like butyrate, acetic acid, and propionic acid [110,111,118]. Thus, SCFAs serve as a critical bridge. One study found that DHA and EPA supplementation significantly alleviated murine hyperglycemia and insulin resistance, improvements mediated by shifts in the gut microbiome and metabolites like SCFAs, glutamate, and bile acids [175]. Consequently, the “gut microbiome–organ axis”—particularly the *n*-3 PUFA–gut–muscle and gut–liver axes—has emerged as a major research hotspot, offering novel metabolic therapeutic targets [176].

Simultaneously, the liver serves as the central metabolic hub for processing these nutrients. *n*-3 PUFA supplementation significantly enhances hepatic glucose transporter and lipoprotein lipase activity [119]. It also improves insulin resistance, reduces hepatic gluconeogenesis and de novo lipogenesis, and increases mitochondrial and fatty acid oxidation-related enzyme activity [98,102,117,118]. Collectively, *n*-3 PUFAs partially alleviate sarcopenia by remodeling liver metabolism, coordinating enzymatic upregulation, and enhancing mitochondrial efficiency.

#### 4.3.3. Combined Interventions

Synergistic strategies combining *n*-3 PUFAs with other nutrients or exercise have become a paramount research focus. For example, combining *n*-3 PUFAs with leucine proves highly effective: formulas incorporating *n*-3 PUFAs, leucine, and specific probiotics (*Lactobacillus paracasei* PS23) successfully delay sarcopenia progression [132], while standard combined interventions significantly improve muscle strength [177].

Similarly, pairing *n*-3 PUFAs with whey protein improves muscle strength and mitigates delayed-onset muscle soreness [112]. Comprehensive multi-nutrient regimens (combining whey protein, creatine, vitamin D, and *n*-3 PUFAs) alongside resistance training yield robust improvements in lean body mass and strength [13].

Integrating *n*-3 PUFAs with specialized physical training offers another potent strategy. Studies combining supplementation with electrical stimulation training [129] or high-intensity interval training plus antioxidant vitamins [17] both report marked benefits for muscle mass and function.

In conclusion, comprehensive strategies merging targeted nutrition with structured exercise define the vanguard of sarcopenia research. Future efforts must elucidate precise synergistic mechanisms to optimize these combined regimens.

#### 4.3.4. Precision Nutrition

Driven by biomarker and genetic insights, precision nutrition is revolutionizing *n*-3 PUFA applications for sarcopenia. As previously established, initial *n*-3 PUFA status dictates supplementation efficacy; therefore, stratifying older adults into “*n*-3-deficient” and “*n*-3-sufficient” cohorts using the Omega-3 Index enables highly tailored and effective interventions [99,100,101].

Genetically, widespread variations in the FADS1 and FADS2 genes heavily influence the endogenous conversion of ALA to EPA and DHA. Individuals carrying “ancestral” haplotypes exhibit poor conversion capacity, necessitating direct preformed LC-PUFA supplementation. Conversely, “derived” haplotype carriers convert more efficiently but risk producing excessive pro-inflammatory metabolites if *n*-6 PUFA intake is high [178]. Consequently, experts advocate incorporating FADS genotyping and baseline fatty acid profiling into routine clinical trial screening to facilitate personalized EPA/DHA dosing regimens [179]. As emphasized by Murphy et al., applying precision nutrition to sarcopenia not only resolves existing literature inconsistencies but substantially elevates intervention efficacy [180]. Ultimately, integrating biomarker stratification with genetic guidance will transition *n*-3 PUFA management from empirical “one-size-fits-all” dosing to individualized precision medicine.

## 5. Limitations and Future Prospects

Despite widespread recognition of their health benefits, *n*-3 PUFAs face challenges regarding inconsistent experimental and clinical evidence. Studies exhibit significant heterogeneity in experimental design, intervention protocols, and outcome indicators, characterized by unstandardized dosages, diverse endpoint definitions, and inadequate baseline assessments. Furthermore, population-level variations in absorption, conversion, and bioavailability remain unresolved.

Based on these limitations, future research should prioritize the following three directions:

First, engineering *n*-3 PUFA derivatives or novel formulations with superior human absorption and bioavailability;

Second, establishing standardized intervention protocols, including clearly defined dosage ranges, administration methods, integrated exercise guidelines, and core outcome sets;

Third, deepening mechanistic research by mapping organ axis regulatory networks (e.g., gut–muscle and muscle–fat axes) and systematically evaluating the comprehensive efficacy of multimodal combined interventions.

## Figures and Tables

**Table 1:** Partial clinical trials.

References	Research Design	Composition and Dosage of *n*-3 Supplements (Daily)	Baseline *n*-3 PUFA Measurement (Yes/No)	Outcome Measures and Overall Effects
Xu, D. [14]	Supplementation Alone RCT 6 month Chinese elderly (>60 years old) Experimental group (n = 97) Control group (n = 90)	fish oil capsules 4000 mg (EPA 1340 mg and DHA 1070 mg)	No	Mixed effect The outcomes were as follows: Thigh circumference: improved TLM: improved ALM: improved Handgrip strength: improved Timed up-and-go test: improved Serum triglycerides: improved HDL-C: improved TC: no change LDL-C: no change
Alkhedhairi, S. A. [130]	Supplementation Alone RCT 6 month Meets NCT04048096 standard 71.2 ± 5.1 years Krill oil group (n = 49, 26 women and 23 men) Placebo group (n = 45, 27 women and 18 men)	krill oil 4000 mg (LCn-3 PUFAs total content 322 mg/g, EPA 193 mg/g, DHA 96 mg/g)	Yes	Mixed effect The outcomes were as follows: Maximal knee extensor torque: improved Handgrip strength: improved Vastus lateralis thickness: improved Erythrocyte EPA content: improved Erythrocyte DHA content: improved *n*-3 index: improved M-wave amplitude: improved Voluntary activation capacity: no change Short physical performance battery: no change Quality of life: no change Other secondary outcomes: no change
Kunz, H. E. [131]	Supplementation Alone RCT 6 month 63 older adults with low activity levels (65–85 years, 29 men and 34 women) *n*-3 PUFAS group (n = 30) Placebo group (n = 33)	capsule 1000 mg (approximately EPA 675 mg, DHA 300 mg) × 4	Yes	Mixed effect The outcomes were as follows: Muscle strength: improved Mitochondrial function: no change Acute exercise response: no change
Da Boit, M. [134]	Supplementation Alone RCT 18 weeks The participants were medically stable, not taking daily painkillers or nutritional supplements, and not participating in any resistance training. (males: n = 27, 70.6 ± 4.5 years; females: n = 23, 70.7 ± 3.3 years) Long chain *n*-3 PUFAs group (n = 23) Placebo group (n = 27)	capsules 1000 mg (EPA 525 mg and DHA 150 mg) × 4	Yes	Mixed effect The outcomes were as follows: Maximal isometric torque (women): improved Maximal isometric torque (men): no change TLM (women): improved TLM (men): no change Maximal isokinetic torque (30, 90, 240°·s^−1^): no change 4 m walk time: no change Standing time: no change Muscle anatomical cross-sectional area: no change Intramuscular fat: no change Plasma triglycerides: improved Blood glucose: no change Insulin: no change Inflammatory markers: no change
Mariangela Rondanelli [132]	Combined Intervention (Leucine and Probiotics LPPS23) RCT, 2 months The patient met the diagnostic criteria for sarcopenia (79.71 ± 4.84 years). Intervention Group (n = 22) Placebo Group (n = 28)	500 mg (64.71% EPA, 29.41% DHA, and 5.88% other *n*-3 PUFAs)	Yes	Positive effect The outcomes were as follows: ALM: improved Tinetti score: improved SPPB total score: improved Handgrip strength: improved VAT: improved Amino acid profile (valine, leucine, isoleucine, and total amino acids): improved
Boutry-Regard [129]	Combined Intervention (whey protein isolate and curcumin softgels) RCT 12 weeks Seniors (60–90 years old) with limited mobility who are living at home CHO (n = 12) WPI (n = 15) WPI + BIO (n = 10)	1500 mg fish oil (18% EPA and 7% DHA)	No	Positive effect The outcomes were as follows: Knee extension strength: improved Walking speed: improved
Nilsson, M. I. [13]	Combined Intervention (Protein, creatine Vitamin and train) RCT 12 weeks Older men with a sedentary or “low-activity” lifestyle and at risk of obesity: M5 (n = 16, 77.4 ± 2.8 years) PLA (n = 16, 74.4 ± 1.3 years)	Fish oil (EPA 1510 mg, DHA 950 mg)	No	Positive effect The outcomes were as follows: ASM: improved TLM: improved ASM/body fat percentage: improved TLM/body fat percentage: improved Maximal strength (handgrip strength; leg press): improved Physical function (5-time chair stand test): improved Quadriceps fast-twitch fiber cross-sectional area (type IIa; type IIx): improved
Dalle, S. [133]	Combined Intervention (Vitamin E and resistance exercise) RCT 14 weeks Healthy, free-living community-dwelling seniors (65–84 years old) *n*-3 group (n = 10) PLAC group (n = 11)	1020 mg (DHA 410 mg, EPA 540 mg) × 3	No	Mixed effect The outcomes were as follows: Isometric knee extensor strength: improved Lower limb push force: no change (both groups increased; time effect) Muscle volume: no change Muscle anabolic signaling (mTORC1): no change C-reactive protein: no change Serum IL-6: trend toward improved
Murphy, C. H. [15]	Combined Intervention (Leucine and Protein) RCT 24 weeks 107 elderly patients at risk of sarcopenia from University College Dublin (UCD), Ireland: CON group (n = 31, 73 ± 7 years) LEU-PRO group (n = 38, 70 ± 5 years) LEU-PRO + ω–3 group (n = 38, 73 ± 6 years)	(EPA 800 mg, DHA 1100 mg) × 2	Yes	Mixed effect The outcomes were as follows: Peak torque during isometric knee flexion: no change Serum triglycerides: improved Total adiponectin: no change HOMA-IR: no change eGFR: improved Cystatin C: improved ALM: no change Handgrip strength: no change Knee extensor strength: no change Physical function: no change Muscle cell apoptosis: no change
Eggimann, A. K. [62]	Combined Intervention (Vitamin D) Large-scale RCT over 3 years. Participants from five European countries were physically active community adults aged 70 and older (n = 1495). Participants were randomly assigned to eight treatment groups, including three interventions: (1) Vitamin D versus Vitamin D placebo control group; (2) *n*-3 fatty acids versus *n*-3 fatty acid placebo control group; (3) Strength training versus attention-controlled exercise emphasizing joint flexibility.	1000 mg (EPA 330 mg, DHA 660 mg)	Yes	Mixed effect The outcomes were as follows: ALMI (change over 3 years): no change ALMI (change at year 1): improved Incident sarcopenia (over 3 years): no change
Félix-Soriano, E [142]	Combined Intervention (train) RCT 16 weeks Women aged 55–70 years, postmenopausal, overweight type II/obese type I P(n = 17) *n*-3(n = 15) P+RT(n = 19) *n*-3 + RT(n = 16)	fish oil concentrate capsules 500 mg (DHA 275 mg and EPA 25 mg) × 6	No	Mixed effect The outcomes were as follows: Body weight and fat mass (declined in all groups, no between-group difference): no change Bone mineral content (strength training vs. non-training): improved Upper limb lean mass (strength training vs. non-training): improved Lower limb fat mass (strength training vs. non-training): improved Muscle strength and quality (strength training vs. non-training): improved Glucose tolerance (strength training vs. non-training): improved Diastolic blood pressure (*n*-3-enriched supplement vs. control): improved Serum triglycerides (*n*-3-enriched supplement vs. control): improved Lower limb muscle mass (*n*-3-enriched supplement vs. control): improved

Footnotes: ALM, Appendicular lean mass; TLM, Total lean mass; ASM/body fat percentage or TLM/body fat percentage, Lean mass-to-fat ratio; ALMI, Appendicular lean mass index; VAT, Visceral adipose tissue; HDL-C, High-density lipoprotein cholesterol; LDL-C, Low-density lipoprotein cholesterol; TC, Total cholesterol; eGFR, Estimated glomerular filtration rate; HOMA-IR, Homeostatic Model Assessment of Insulin Resistance.

## Data Availability

No new data were created or analyzed in this study. Data sharing is not applicable to this article.
